# Supplementary material for: The causal relationship between the human gut microbiota and pyogenic arthritis: a Mendelian randomization study
Source: Front Cell Infect Microbiol. 2024 Nov 26;14:1452480. doi: 10.3389/fcimb.2024.1452480 (PMC11629706; doi:10.3389/fcimb.2024.1452480)
Supplement: Supplementary file 1 [file DataSheet1.zip › mendelian test/ebi-a-GCST90027614.csv_forest.pdf]

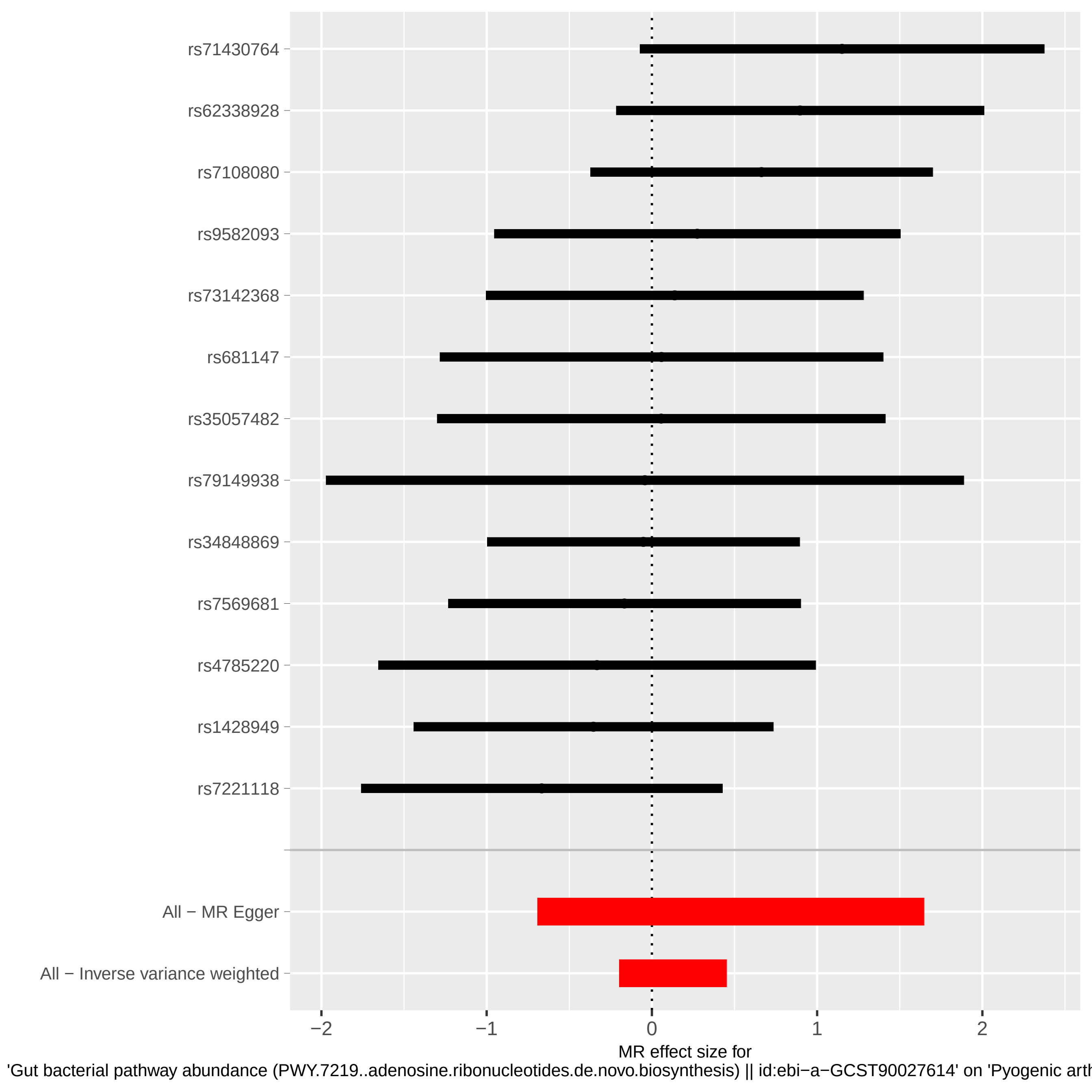
rs71430764
rs62338928
rs7108080
rs9582093
rs73142368
rs681147
rs35057482
rs79149938
rs34848869
rs7569681
rs4785220
rs1428949
rs7221118
All − MR Egger
All − Inverse variance weighted
−2
−1
0
1
2
MR effect size for
'Gut bacterial pathway abundance (PWY.7219..adenosine.ribonucleotides.de.novo.biosynthesis) || id:ebi−a−GCST90027614' on 'Pyogenic arth
